# Navigating geographical disparities: access to obstetric hospitals in maternity care deserts and across the United States

**DOI:** 10.1186/s12884-024-06535-7

**Published:** 2024-05-08

**Authors:** Jazmin Fontenot, Christina Brigance, Ripley Lucas, Ashley Stoneburner

**Affiliations:** Perinatal Data Center, March of Dimes, 1550 Crystal Drive Suite 1300, Arlington, VA USA

**Keywords:** Maternity care deserts, Maternal health, Disparities in maternity care access, Spatial analysis

## Abstract

**Background:**

Access to maternity care in the U.S. remains inequitable, impacting over two million women in maternity care “deserts." Living in these areas, exacerbated by hospital closures and workforce shortages, heightens the risks of pregnancy-related complications, particularly in rural regions. This study investigates travel distances and time to obstetric hospitals, emphasizing disparities faced by those in maternity care deserts and rural areas, while also exploring variances across races and ethnicities.

**Methods:**

The research adopted a retrospective secondary data analysis, utilizing the American Hospital Association and Centers for Medicaid and Medicare Provider of Services Files to classify obstetric hospitals. The study population included census tract estimates of birthing individuals sourced from the U.S. Census Bureau's 2017-2021 American Community Survey. Using ArcGIS Pro Network Analyst, drive time and distance calculations to the nearest obstetric hospital were conducted. Furthermore, Hot Spot Analysis was employed to identify areas displaying significant spatial clusters of high and low travel distances.

**Results:**

The mean travel distance and time to the nearest obstetric facility was 8.3 miles and 14.1 minutes. The mean travel distance for maternity care deserts and rural counties was 28.1 and 17.3 miles, respectively. While birthing people living in rural maternity care deserts had the highest average travel distance overall (33.4 miles), those living in urban maternity care deserts also experienced inequities in travel distance (25.0 miles). States with hotspots indicating significantly higher travel distances included: Montana, North Dakota, South Dakota, and Nebraska. Census tracts where the predominant race is American Indian/Alaska Native (AIAN) had the highest travel distance and time compared to those of all other predominant races/ethnicities.

**Conclusions:**

Our study revealed significant disparities in obstetric hospital access, especially affecting birthing individuals in maternity care deserts, rural counties, and communities predominantly composed of AIAN individuals, resulting in extended travel distances and times. To rectify these inequities, sustained investment in the obstetric workforce and implementation of innovative programs are imperative, specifically targeting improved access in maternity care deserts as a priority area within healthcare policy and practice.

**Supplementary Information:**

The online version contains supplementary material available at 10.1186/s12884-024-06535-7.

## Background

Each year in the United States, an estimated 60,000 birthing people experience unexpected outcomes during labor and delivery, or severe maternal morbidities, which have both short- and long-term impacts on their health [1]. Additionally, approximately 750 women die from pregnancy-related deaths [1]. Complications and deaths from pregnancy and childbirth are largely preventable [1, 2], indicating ample opportunity for improvement. Access to healthcare is critical during the perinatal period; however, hospitals that offer maternity care services are not equitably distributed across the country. Further, access to maternity care depends on several other factors, including the availability of obstetric providers, risk-appropriate care, and health insurance [3–5].

More than 2 million women of childbearing age live in maternity care “deserts” 1 [6], defined as counties without birthing facilities or maternity care providers [7]. Areas of inadequate access to maternity care are created through systems and policies that deplete community resources. Hospital and maternity unit closures [8, 9], obstetric workforce shortages [8, 9], inadequate Medicaid reimbursement rates [9, 10], and systemic racism and classism [11], have contributed to the increase in more counties with low or no access to care. One-third of all U.S. counties are maternity care deserts, and 60% are in rural areas [7]. Maternity care deserts are associated with an increased risk of pregnancy-related death up to one year postpartum [12]. Birthing people living in rural areas have a 9% greater risk of severe maternal morbidity and mortality from pregnancy and childbirth and are more likely to report difficulties in accessing quality care compared to urban residents [13–16].

Across the nation, the closure of obstetric hospitals has played a role in the rise of maternity care deserts, resulting in increased distance to care [5, 7]. These extended distances not only discourage the use of preventive care but also impact the overall health and quality of care for individuals during pregnancy and childbirth [17, 18]. The period from early prenatal through late postpartum often necessitates multiple trips to obstetric hospitals to access care, particularly for hospitals that offer comprehensive services from pediatricians, midwives, obstetricians, and family physicians at a single location [3]. In addition to the financial strain and heightened stress and anxiety with increased distance to care [19], the risk of adverse maternal outcomes and neonatal intensive care unit (NICU) admission has been shown to increase with longer travel distances and time to care, even after adjusting for patient characteristics, pregnancy risk, rurality, neighborhood characteristics and delivery hospital NICU acuity [20]. Unlike established standards for reaching a hospital promptly in medical emergencies like stroke [21], there is currently no standardized guideline for the travel time to reach a hospital during an obstetric emergency or for prenatal and postpartum care [22, 23].

### Study purpose

This research aims to assess access to obstetric hospitals across the U.S., focusing on inequities for birthing people in maternity care deserts and rural areas. A secondary purpose is to highlight the differing distance and time to obstetric hospitals by the predominant race/ethnicity in each census tract. Using spatial analysis techniques, our study maps the typical travel distance and time needed to reach the nearest obstetric hospital in the U.S.

## Methods

### Research design

We conducted a retrospective analysis of secondary data to estimate the travel distance and time to obstetric hospitals across the U.S. The main outcomes were geographic distance, in miles, and drive time, in minutes, from residential census tracts to the nearest hospital that provided obstetric care. Analyses were cross-sectional and estimated travel distances for all birthing people in the U.S. and used census tract fertility data and 2021 hospital availability. We examined differences in driving distance and driving time by maternity care desert designation, rurality, and predominant race/ethnicity.

### Data sources

#### Births

This analysis used five-year fertility estimates from the U.S. Census Bureau’s 2017-2021 American Community Survey (ACS) [24]. The ACS estimates the number of people who reported giving birth in the past year for all U.S. census tracts. At the time of the analysis, 98% of births occur in a hospital setting [25]. Demographic variables from the ACS were used to analyze distance by the predominant race/ethnicity in each census tract. Data on race and ethnicity in the ACS are based on self-identification by the respondent and collapsed into the following categories: White, Black, Asian, Native Hawaiian or other Pacific Islander, American Indian or Alaska Native, and Hispanic or Latino [26]. Predominant race/ethnicity categories were determined by calculating the percent of all race/ethnicity categories for the population of each census tract and assigning the race/ethnicity with the highest percentage.

#### Census Tracts

The spatial analysis files included point centroid locations for U.S. census tracts linked with ACS birth estimates. Point centroid locations were spatially weighted to account for population density within each census tract using data from the IPUMS National Historical Geographic Information System (NHGIS) [27]. U.S. Census Topologically Integrated Geographic Encoding and Referencing (TIGER/Line) shapefiles were used for data visualization at the county and census tract level [28].

#### Hospitals

**Fig. 1:**
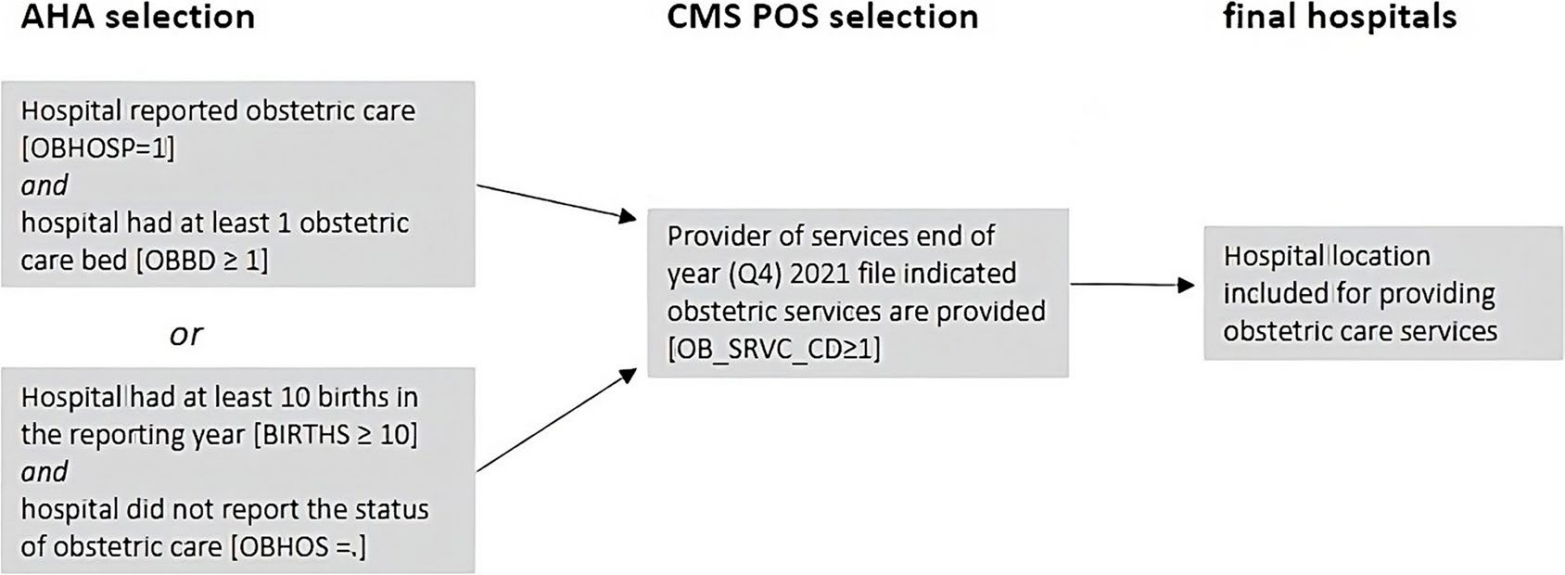
Flow Diagram for Obstetric Hospital Validation

Hospital location data were obtained from the American Hospital Association (AHA) 2021 Survey [29]. The AHA Annual Survey provides data for more than 6,200 hospitals and healthcare systems, including Indian Health Service (IHS) hospitals, and contains addresses for geocoding. The use of AHA data is consistent with federal government agencies as the most comprehensive hospital data source for health research [30]. The AHA survey uses self-reported data to classify hospitals with obstetric care services. To check for missing obstetric hospitals, we performed a secondary validation of AHA hospital locations using the Centers for Medicare and Medicaid (CMS) Provider of Service (POS) files for 2021 and followed enhanced methods of identification described by prior research [31, 32]. Only hospitals that listed providing obstetric services according to CMS POS data (OB_SRV_CD>=1) were included. Obstetric hospitals were selected if they met the following criteria (see Fig. 1).

#### Designations for maternity care access

Maternity care access designations were created by the Perinatal Data Center at March of Dimes [7, 33]. U.S. counties were previously classified using data from the Human Resources Service Administration’s (HRSA) Area Health Resource Files (2021-2022) for obstetric providers (OB-GYNs, certified midwives and certified nurse midwives) and obstetric hospitals; counts of family physicians who deliver babies from the American Board of Family Medicine (2017-2020); birth estimates from the National Center for Health Statistics (NCHS) 2021 natality data; and birth center data from the 2021 American Association of Birth Centers (AABC) [25, 34–36]. For this analysis, we combined the low and moderate maternity care access categories into one “limited” access designation. Levels of maternity care access are defined as follows:

- Maternity care desert: A county with no hospitals providing obstetric care, no birth centers, and no obstetric providers, which include OB-GYNs, certified nurse midwives, certified midwives, and family physicians who reported delivering babies.
- Limited access: A county with fewer than two hospitals or birth centers offering obstetric care and fewer than 60 obstetric providers per 10,000 births.
- Full access: A county with two or more hospitals or birth centers offering obstetric services or more than 60 obstetric providers per 10,000 births.

#### Rural areas

Rural-Urban Continuum codes, developed by the U.S. Department of Agriculture, Economic Research Service, classify counties into nine categories by population size based on census-defined urbanized areas and by adjacency to metropolitan areas [37]. The categories can be further classified as metropolitan/nonmetropolitan or urban/rural. Metropolitan categories were defined as a county with a metropolitan area of 1 million people (about the population of Delaware) or more, 250,000 to 1 million, or fewer than 250,000 (one, two, or three on the Rural-Urban Continuum). All the other categories (four or more on the Rural-Urban Continuum) are considered nonmetropolitan. Urban areas include all metropolitan areas and/or areas adjacent to a metropolitan area with a population greater than 2,500 (one through four and six on the Rural-Urban Continuum). Rural includes areas with a population of 2,500 or more not adjacent to a metropolitan area and areas with fewer than 2,500 people (five and seven or higher on the Rural-Urban Continuum).

### Analyses

ArcGIS Pro, version 3.0 [38], was used to geocode validated hospital locations. Linked ACS birth data with population-weighted census tract point locations were used as the residential incidents (starting point locations for GIS calculations). The ESRI Network Analyst Extension Closest Facility Solver [39] calculated the driving time and mileage distance from each birth to the nearest obstetric hospital location. ArcGIS Online network data was used for routing services, a regularly maintained database of comprehensive street data that includes historical, live, and predictive road networks. To increase the generalizability of our results and because travel to obstetric hospitals can occur at any time of the day, we did not account for fluctuations in traffic conditions.

All the statistical analyses were performed using SAS software, version 9.4 [40]. Imported GIS census tract calculations were aggregated at the county-level for comparison with county-level maternity care access designations, rurality, metropolitan status, and predominant race/ethnicity. We tested significant differences in travel time by county-level factors using a one-way ANOVA test for factors with three or more levels (maternity care access designation) and t-tests for all others. Travel time cutoffs of 30 and 60 minutes were used to describe the percentage of birthing people who live far from obstetric care. We report means and standard deviations rather than medians to highlight the skewing of data in maternity care deserts, which would otherwise be considered outliers.

We aimed to determine if specific counties exhibited notably higher or lower travel distances to obstetric hospitals compared to their surrounding areas. To identify areas with statistically significant variations in travel distances, we conducted a hotspot analysis for the continental U.S., also known as Getis-Ord Gi*, using ArcGIS Pro [41]. This approach allowed us to pinpoint geographic areas where travel distances to obstetric hospitals were significantly higher (referred to as “hot spots”) or lower (referred to as “cold spots”), to provide insight into regional disparities in access to obstetric care. Counties with 10 or fewer births were suppressed from the analysis to ensure statistical reliability.

## Results

We identified 3,991,060 birthing people among 85,396 census tracts across the U.S., D.C., and Puerto Rico and 2,630 hospitals that provide obstetric care. Obstetric hospitals were geocoded with a match score of 99.0%; nine hospitals did not have a match and were manually reviewed using Google Maps to obtain point location data. Four hospitals were excluded after manual review yielded no location data. Drive-time routes were calculated for 99.6% of the total estimated births; eight census tracts did not have calculable road network routes, accounting for less than 0.4% of all estimated births.

### Distance overall

**Fig. 2:**
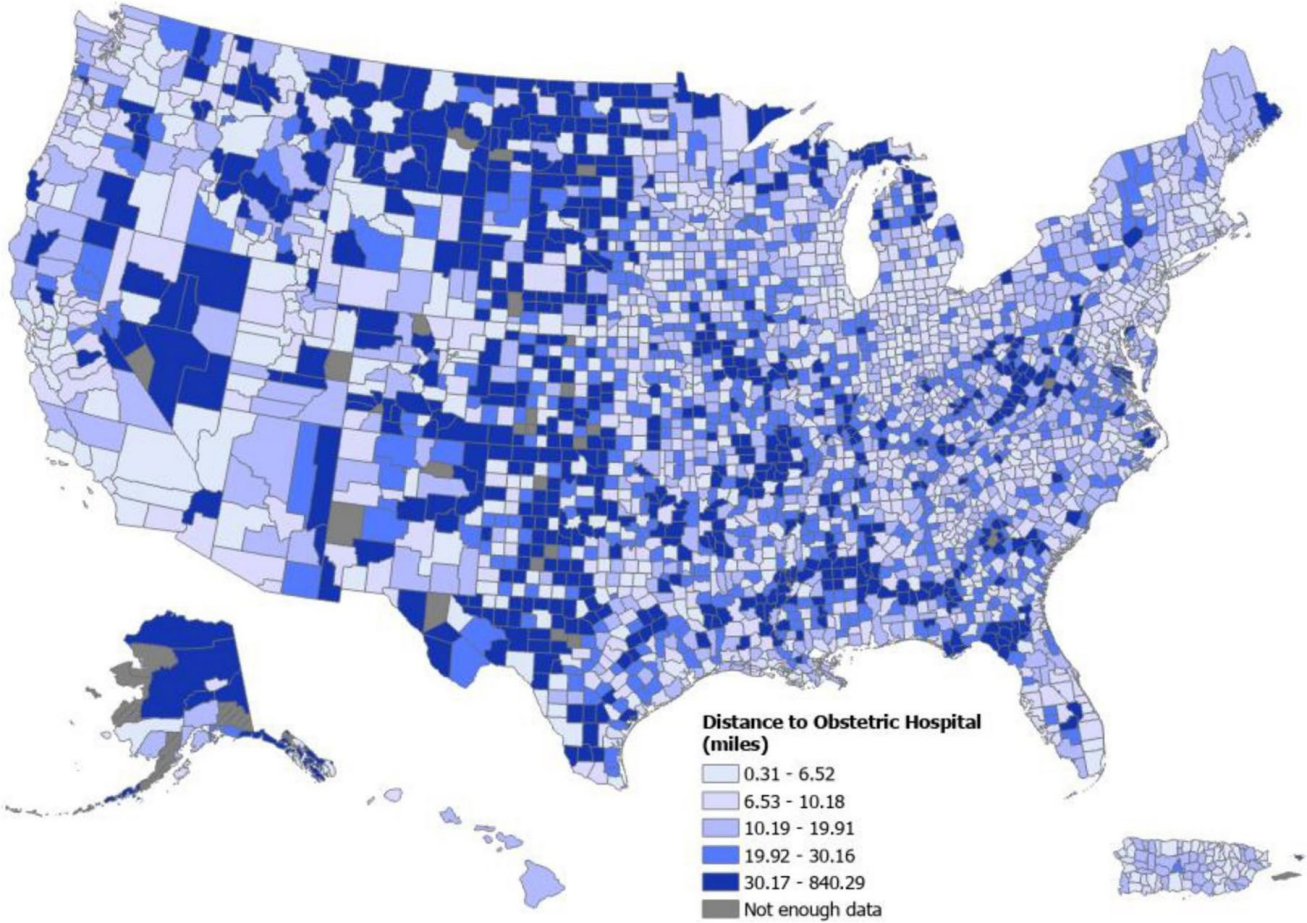
Distribution of Travel Distance to Nearest Obstetric Hospital in the U.S. by County

Figure 2 displays the quintile distribution of mean travel distance, in miles, by county across the U.S. The mean distance and time to the nearest obstetric hospital were 8.3 miles (SD 9.0) and 14.1 minutes (SD 21.8), respectively (Table 1). Nearly all of the U.S. population lived within 1 hour of the nearest obstetric care hospital (99.7%), and 93.6% lived within 30 minutes.

**Table 1:** Mean miles and minutes to nearest obstetric hospital by census tract

	**Births N**	**Mean distance miles (SD)**	**Mean time minutes (SD)**
**US Census tracts**	3,991,060	8.3 (9.0)	14.1 (21.8)
**MCD Designation**			
Maternity care desert	145,146	28.1 (21.2)	36.5 (102.3)
Limited access	248,347	14.2 (10.8)	20.1 (11.8)
Full access	3,597,567	7.1 (6.6)	12.7 (8.5)
**Rurality**			
Urban	3,785,735	7.8 (7.4)	13.5 (8.6)
Rural	205,325	17.3 (21.9)	24.6 (88.1)
**Metropolitan Residence**			
Nonmetro	536,349	16.0 (16.8)	22.3 (55.6)
Metro	3,454,711	7.1 (6.3)	12.8 (7.5)
**Predominant race/ethnicity**			
White	2,763,765	9.2 (8.8)	15.0 (11.0)
Hispanic	713,750	6.1 (6.7)	11.3 (7.5)
Black	406,972	6.3 (6.5)	11.9 (7.4)
Asian	92,886	5.2 (18.3)	12.5 (107.7)
AIAN	9,772	27.7 (41.3)	45.9 (203.3)
Multiracial	1,967	12.8 (10.8)	23.4 (18.5)
NH/PI	1,360	17.2 (10.5)	26.4 (14.2)
**Travel Time to Closest Obstetric Care (%)**			
<=15 minutes	2,692,070	-	67.5%
<=30 minutes	3,736,131	-	93.6%
<=45 minutes	3,933,929	-	98.6%
<=60 minutes	3,977,229	-	99.7%

### Distance by maternity care designation

The mean time and distance to the closest obstetric hospital increased as access to care decreased. The mean distance and time to care by maternity care access designation was 7.1 miles or 12.7 minutes for full access areas; 14.2 miles or 20.1 minutes for limited access areas; and 28.1 miles or 36.5 minutes in maternity care deserts. The mean travel time among birthing people in maternity care deserts was 3.9 times greater than in full-access areas.

### Distance by rurality

The travel distance and time to care for birthing people living in rural and urban areas was 17.3 miles or 24.6 minutes and 7.8 miles or 13.5 minutes, respectively. Differences in travel distance and time were also observed when comparing metropolitan (7.1 miles or 12.8 minutes) to nonmetropolitan (16.0 miles or 22.3 minutes) areas. When examining rural and urban distance differences by maternity care access designation, we found that distances were similar for limited-access areas but were greater regardless of rural designation for maternity care deserts (Table 2). Those living in rural maternity care deserts traveled 1.9 times farther than the average birthing person in a rural area and 4.0 times farther than the average birthing person overall. In urban maternity care deserts, the average travel distance was 3.2 times farther than the average person living in an urban area and 3.0 times farther than the average birthing person. These discrepancies highlight that even in urban areas, those living in maternity care deserts travel farther than those living in rural areas.

**Table 2:** Distance and time by geographic characteristics and maternity care designation

	**Full access**		**Limited access**		**Maternity care desert**	
	**Miles**	**Time**	**Miles**	**Time**	**Miles**	**Time**
**Overall**	7.1	12.7	14.2	20.1	28.1	36.5
**Rurality**						
Urban	6.9 (6.1)*	12.6 (7.5)*	14.2 (10.6)*	20.1 (11.5)*	25.0 (11.2)*	31.4 (11.9)*
Rural	11.4 (13.6)*	17.1 (20.1)*	14.6 (13.7)*	19.8 (15.1)*	33.4 (31.3)*	45.4 (169.4)*
**Predominate race/ethnicity**						
White	7.8 (6.9)*	13.5 (8.3)*	14.9 (10.5)*	20.9 (11.6)*	28.0 (13.4)*	34.8 (23.8)*
Hispanic	5.6 (5.5)*	10.9 (6.6)*	10.7 (11.5)*	15.7 (11.9)*	24.2 (17.2)*	29.3 (16.1)*
Black	5.3 (4.4)*	10.9 (5.5)*	10.7 (10.8)*	16.3 (11.7)*	27.6 (10.7)*	33.2 (11.7)*
Asian	4.8 (3.4)*	10.2 (4.9)*	--	--	--	--
AIAN	24.7 (27.7)*	35.8 (58.0)*	26.2 (19.5)*	31.4 (20.7)*	59.0 (105.3)*	161.1 (657.3)*

^*^p-value<0.001

### Distance by predominant race

Census tracts classified as predominantly American Indian and Alaska Native (AIAN) had the highest travel distance and time to obstetric hospitals. On average, those living in predominantly AIAN census tracts travel 27.8 miles or 45.9 minutes to their nearest obstetric hospital, 3.3 times farther than the average travel time in the U.S. Driving distance and time differences are exacerbated for predominantly AIAN census tracts withinmaternity care deserts, where the closest obstetric hospital is 59.0 miles or 161.1 minutes away, on average. This disparity is 2.0 times greater than that of all other races and ethnicities in maternity care deserts. Predominantly AIAN census tracts are 2.2 times more likely to be in maternity care deserts than census tracts with predominantly White residents.

### Travel distance by state and hot spot analysis

The travel times and distances by state are shown in more detail in Supplementary File 1. The states with the highest overall travel distances were Alaska, West Virginia, Montana, Mississippi, and South Dakota. The states with the highest travel times included Hawaii and North Dakota. The states with the lowest overall travel distances were D.C., New Jersey, Connecticut, New York, Rhode Island and California, all with mean travel distances lower than six miles.

**Fig. 3:**
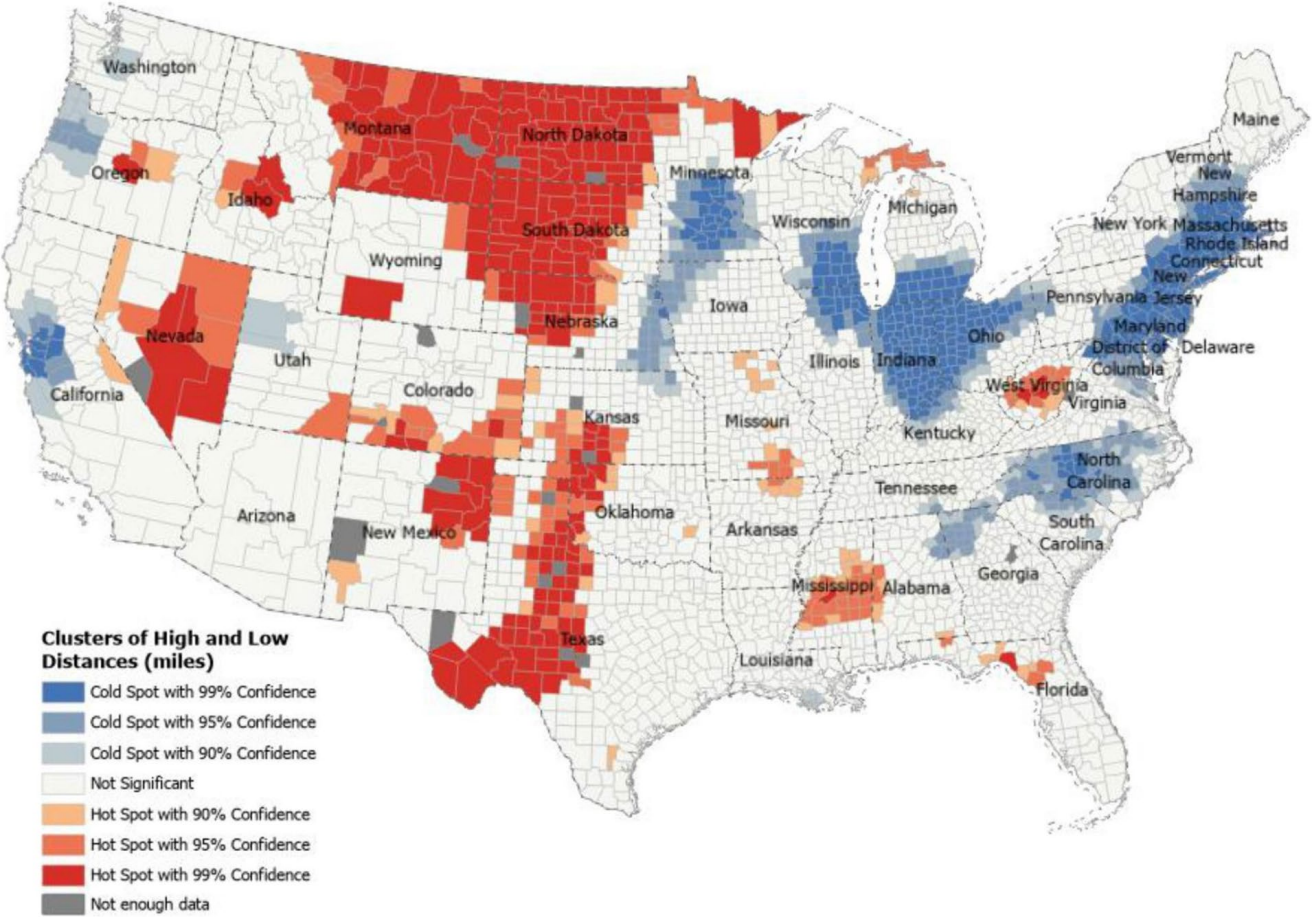
Hot Spot Analysis for Low and High Travel Distance to Nearest Obstetric Hospital

Hot spot analysis revealed areas with statistically significant high and low travel distances in the continental U.S. (Fig. 3). From this analysis, we found that regions of Montana, South Dakota, North Dakota, and Nebraska all had the highest concentrations of maternity care deserts, and these were also areas with statistically significant spatial clustering (hot spots) for high travel distances to obstetric care. States with large populations living in maternity care deserts also had statistically significant spatial clustering, indicating long travel distances; these included Texas, Mississippi, Oklahoma, and Missouri. Areas for low travel times across the U.S. were concentrated in the northeast and included states with predominantly metropolitan areas such as D.C., Rhode Island, and New Jersey. Additional clusters of low travel times were found for the East North Central region of the U.S., Northern California, Minnesota, and North Carolina (Fig. 3).

## Discussion

Consistent with the findings of prior literature, we found that most of the U.S. population lived within one hour of their nearest obstetric hospital [15, 42–47]. Our analysis revealed that nearly 94% of the birthing population in the U.S. lived within 30 minutes of an obstetric hospital; however, this percentage decreased to 86% among the birthing population who lived in maternity care deserts. Although the estimated mean travel distance and time to reach the nearest hospital with obstetric services were relatively low (8.3 miles and 14.1 minutes), this study is the first to characterize the geographic accessibility of maternity care deserts.

Birthing people living in maternity care deserts traveled nearly four times farther to reach their closest obstetric hospital than those living in full-access counties (28.1 miles vs. 7.1 miles). In some states, this difference exceeded 40 miles. It is well documented that healthcare access is limited in rural areas; however, our analysis further highlights access barriers for people living in maternity care deserts in urban areas (40% of all classified maternity care deserts). In contrast to the mean travel distance for those living in urban counties (7.8 miles) and rural counties (17.3 miles), the mean travel distance in an urban maternity care desert was 25.0 miles, a difference of 3.2 and 1.5 times farther, respectively. These findings highlight that living in a maternity care desert, whether urban or rural, significantly impacts travel distance to the nearest obstetric hospital. Given the relationship between poor maternal health outcomes and living in a rural area [12–14], further research is necessary to assess health outcomes among birthing people living in maternity care deserts.

Analysis of mean travel distance and time to the closest obstetric hospital does not account for additional barriers that birthing people in areas of no or low access may face to reach risk-appropriate care. Apart from the journey required at the time of labor, individuals may need to travel multiple times to an obstetric hospital for prenatal, postpartum, or specialty care. Higher level care is typically available in high-volume hospitals with greater resources, including NICUs and specialized staff better equipped to handle rare maternal and infant complications. Studies have shown that maternal and infant outcomes are better in hospitals with high birth volumes than those with low birth volumes. For example, infant survival is greater in high-volume hospitals for both high- and low-risk infants [48]. Additionally, the risk of severe maternal morbidity is greater among obstetric patients who deliver at lower-volume hospitals in rural areas [19, 44]. High-volume hospitals are often located in metropolitan areas where most infants are delivered. In contrast, high-volume hospitals account for only 10% of all obstetric hospitals in rural areas where less than 20% of infants born had a high-volume hospital within 30 miles [44]. Future research should quantify barriers faced by birthing people living in maternity care deserts when seeking more comprehensive care, either by choice or necessity.

Our findings were consistent with others, which found disparities in travel distance by race/ethnicity [49–51]. For birthing people living in predominantly AIAN census tracts that are located within maternity care deserts, the mean distance to reach obstetric care was 59.0 miles, 2.1 times farther than the distance traveled by those living in predominantly White census tracts in maternity care deserts. We found that, regardless of maternity care access designation, those living in predominantly AIAN census tracts travel the farthest to reach obstetric care compared to birthing people living in all other census tracts. Travel distance is exacerbated for birthing people living in rural areas and on American Indian reservations, where access is limited, and bypassing the nearest hospital to give birth is more common and necessary for risk-appropriate care [49]. States identified in our hot spot analysis for statistically high travel distances to care were overwhelmingly concentrated in areas with high AIAN populations compared to other U.S. states [Additional file 1]. AIANs are two times more likely to die from pregnancy complications than White mothers and Indigenous people living in rural areas have the highest rates of severe maternal morbidity and mortality [50, 51]. These findings highlight the need to address inequities and implement policies that support maternity care for AIAN communities with barriers in distance and time to care.

### Strengths and limitations

There are several limitations to note in this study. The analysis determined the predominant race/ethnicity within each census tract, achieved by considering all race/ethnicities present and categorizing them based on the most prevalent race/ethnicity. When comparing these categories to average distances, we found that census tracts predominantly comprised of AIAN populations had the longest travel times and distances to the nearest obstetric hospital. It is important to note that these distances are high even though it encompasses Indian Health Service hospitals, which provide care to these populations. However, it is crucial to acknowledge that these distances are not exact representations based on the race/ethnicity of the birthing person, but rather reflect averages within the census tract, which may predominantly consist of a particular race/ethnicity.

We analyzed driving time and distance to the nearest obstetric hospital in the U.S.; however, the average birthing person may bypass their closest obstetric hospital to receive more comprehensive or better-quality care. In some circumstances, insurance coverage may not extend past a birthing person’s state of residence, and several of the closest points of care in our analysis included obstetric hospitals in states that crossed residential borders. Additionally, we report mean travel distance and travel time rather than the median to highlight the skewing of data for those who live in maternity care deserts, which in other analytical studies would be considered outliers. Median travel distance (5.6 miles) and time (11.6 minutes) are lower than means, largely due to the majority of the population living in metropolitan areas where the vast majority of hospitals are located. We did not specify day start or stop times to account for fluctuations in traffic conditions or weather seasonality where driving conditions could impact travel time. GIS analyses of drive times were based on car transport calculations and are not generalizable for bus or public transit travel. Due to these limitations, in addition to using census tract weighted point locations rather than patient addresses, the results likely underestimate the actual travel distance and time to reach obstetric care.

Despite these limitations, our results are derived from extensive and validated datasets and are generalizable to hospital deliveries, accounting for 98% of all U.S. births in 2022 [25]. In addition, response rates of the AHA hospital data vary across states and health systems; however, validation using CMS data allowed for accurate identification of hospitals with obstetric care available across the nation. Our GIS analysis used population-weighted centroid locations to account for where the majority of birthing people reside in each census tract. Census tract centroids allowed for greater granularity in calculations of travel distance and time. ArcGIS Pro Network Analyst Extension allowed us to obtain the shortest driving distance and times to care using live data for streets, railroads, and ferries. The use of transport network analysis enabled us to model real-time world phenomena in road travel and is the recommended method to estimate geographic accessibility instead of using straight-line Euclidian distance [43, 52–54].

### Implications

A lack of access to maternity care is a complicated issue that requires innovative and targeted solutions. While the mean distance and time to care is generally low in much of the U.S., significant barriers persist, particularly for those in maternity care deserts. Essential to addressing these challenges is continued investment in healthcare infrastructure, including incentives to maintain and train clinicians in these areas. Increasing the obstetric workforce, not only in number but also in geographic distribution and racial/ethnic diversity, is essential to meet the needs of the U.S. birthing population. One effective approach is supporting and expanding midwifery services, which have been shown to improve outcomes, increase culturally appropriate care, and lower costs of obstetric care [55, 56]. Additional efforts should focus on increasing reimbursement rates for midwifery care, eliminating cumbersome licensing requirements, and addressing hospital resistance to employing midwives [57].

The White House Blueprint for Addressing the Maternal Health Crisis outlines several goals that target improvements in access for rural communities and investments in the maternal health workforce [57]. Notably, the HRSA-funded Rural Maternity and Obstetrics Management Strategies (RMOMS) program has expanded access in communities across 9 states, connected high-risk pregnant women to maternal-fetal medicine specialists, and facilitated hiring new providers. [58] Furthermore, HRSA’s Maternity Care Target Areas (MCTA) guide the optimal placement of obstetricians and certified nurse midwives within the National Health Service Corps [59].

Telehealth, which includes virtual visits, remote patient monitoring, mobile healthcare, and real-time telemedicine interactions between patients and providers, has proven effective in mitigating obstetric provider shortages, particularly in rural areas with limited access to specialty care [60]. Supporting innovative telehealth initiatives ensures equitable access to obstetric care, regardless of geographical location.

Despite having a limited impact on obstetric unit closures [61], policies such as Medicaid extension and expansion have shown positive effects on birth and maternal health outcomes for individuals in poverty [62]. Policymakers should consider expanding Medicaid coverage in all states to mitigate the travel burden for individuals with low income, ensuring access to a broader range of potential hospitals offering obstetric care. Policymakers and hospital administrators must also consider the implications of closures on travel burdens for individuals in urban and rural maternity care deserts. Understanding these implications is essential for crafting effective policies and interventions to address these challenges.

While access to healthcare should be a human right [63], this study shows that where a person lives greatly impacts the ability to access maternity care. Not only are maternity care deserts lacking the obstetric care facilities and providers needed to care for birthing people, living in these areas has a fourfold impact on the time and distance to reach maternity care. This study adds to extensive research that demonstrates inequities in access to maternity care across the U.S., which are created and perpetuated through the failure of our policies and systems. To enact change, we must address the underlying systemic issues that persist.

## Conclusion

Our findings revealed disparities in access to obstetric hospitals, for birthing individuals residing in maternity care deserts, rural areas, and predominantly AIAN census tracts. These findings highlight the importance of finding solutions to support populations that reside farther away from maternity care to reduce the risk of adverse outcomes associated with extended travel during pregnancy, childbirth and postpartum. To mitigate these disparities, sustained investment in the obstetric workforce is crucial, along with implementing innovative practices and programs to expand access, especially in maternity care deserts. Addressing systemic inequities demands a multifaceted, multi-sectoral approach that prioritizes healthcare access as a fundamental right and actively dismantles disparities in obstetric care nationwide.

### Supplementary Information

**Supplementary Material 1.**

## Data Availability

This material is based upon data provided by the American Board of Family Medicine (ABFM). The views expressed herein are those of the authors and do not necessarily reflect the position of policy of ABFM. The data supporting this study’s findings are available from the American Hospital Association (AHA), but restrictions apply to the availability of these data, which were used under license for the current research and so are not publicly available. Data are, however, available from the authors upon reasonable request and with permission of AHA.

## Abbreviations

AIAN: American Indian/Alaska Native
AHA: American Hospital Association
ESRI: Environmental Systems Research Institute
GIS: Geographic Information Systems
HRSA: Health Resources and Services Administration
MCTA: Maternity Care Target Area
NH/PI: Native Hawaiian/Pacific Islander
OB-GYN: Obstetrician-Gynecologist
U.S.: United States

## Footnotes

1 We recognize that using the term desert to describe counties is limited and can oversimplify the complex issues faced by communities most impacted by the maternity care crisis. For purposes of this paper “maternity care desert” is used to align with prior March of Dimes reports, we use the term desert to refer to a quality or ability to fail (someone), especially at a crucial moment when most needed, in the context of maternity care availability.

## References

[1] Declercq E, Zephyrin LC. Severe Maternal Morbidity in the United States: A Primer. Commonwealth Fund. 2021. https://www.commonwealthfund.org/publications/issue-briefs/2021/oct/severe-maternal-morbidity-united-states-primer. Accessed 31 Mar 2024.

[2] Centers for Disease Control and Prevention (CDC). Four in 5 pregnancy-related deaths in the U.S. are preventable. https://www.cdc.gov/media/releases/2022/p0919-pregnancy-related-deaths.html (accessed 14 Dec. 2023).

[3] Backes EP, Scrimshaw S, National Academies of Sciences E and M. Birth settings in America: outcomes, quality, access, and choice. Washington, DC: National Academies Press; 2020. 32049472

[4] Eliason EL, Daw JR, Allen HL (2021). Association of Medicaid vs Marketplace Eligibility on Maternal Coverage and Access with Prenatal and Postpartum Care. JAMA Netw Open.

[5] Brigance C, Lucas R, Jones E, Davis A, Oinuma M, Mishkin K, et al. Nowhere to Go: Maternity Care Deserts Across the U.S. (Report No. 3). 2022. https://www.marchofdimes.org/sites/default/files/2022-10/2022_Maternity_Care_Report.pdf.

[6] Kozhimannil KB (2023). Declining access to US maternity care is a systemic injustice. BMJ.

[7] Fontenot J, Lucas R, Stoneburner A, Brigance C, Hubbard K, Jones E, et al. Where You Live Matters: Maternity Care Deserts and the Crisis of Access and Equity in [All States]. 2023. https://www.marchofdimes.org/peristats/reports/alabama/maternity-care-deserts.

[8] Kramer  KJ, Elena Rhoads-Baeza M, Sadek S, Chao CR, Bell C, Recanati MA (2022). Trends and Evolution in Women’s Health Workforce in the First Quarter of the 21st Century. World J Gynecol Womens Health.

[9] Hung P, Kozhimannil KB, Casey MM, Moscovice IS (2016). Why Are Obstetric Units in Rural Hospitals Closing Their Doors?. Health Serv Res.

[10] Carroll C, Planey A, Kozhimannil KB (2022). Reimagining and reinvesting in rural hospital markets. Health Serv Res.

[11] McGregor AJ, Hung P, Garman D, Amutah-Onukagha N, Cooper JA (2021). Obstetrical unit closures and racial and ethnic differences in severe maternal morbidity in the state of New Jersey. Am J Obstet Gynecol MFM.

[12] Wallace M, Dyer L, Felker-Kantor E, Benno J, Vilda D, Harville E (2021). Maternity Care Deserts and Pregnancy-Associated Mortality in Louisiana. Women’s Health Issues.

[13] Kozhimannil KB, Interrante JD, Henning-Smith C, Admon LK (2019). Rural-Urban Differences in Severe Maternal Morbidity and Mortality in the US, 2007–15. Health Aff (Millwood).

[14] Hostetter M, Klein S. Restoring Access to Maternity Care in Rural America. Commonwealth Fund. 2021. https://www.commonwealthfund.org/publications/2021/sep/restoring-access-maternity-care-rural-america. Accessed 24 Jan 2024.

[15] Parker K, Horowitz JM, Brown A, Fry R, Cohn D, Igielnk R. What Unites and Divides Urban, Suburban and Rural Communities. Pew Research Center. 2018. https://www.pewresearch.org/wp-content/uploads/sites/20/2018/05/Pew-Research-Center-Community-Type-Full-Report-FINAL.pdf.

[16] Lam O, Broderick B, Toor S. How far Americans live from the closest hospital differs by community type. Pew Research Center. 2018. https://www.pewresearch.org/short-reads/2018/12/12/how-far-americans-live-from-the-closest-hospital-differs-by-community-type/#:~:text=Overall%2C%2018%25%20of%20Americans%20live,areas%20than%20in%20rural%20ones.

[17] Care Institute of Medicine (US) Committee to Study Outreach for Prenatal. Barriers to the Use of Prenatal Care. In: Brown SS, editor. Prenatal Care: Reaching Mothers, Reaching Infants., National Academies Press (US); 1988. https://www.ncbi.nlm.nih.gov/books/NBK217704/. 25032444

[18] DiPietroMager NA, Zollinger TW, Turman JE, Zhang J, Dixon BE. Routine Healthcare Utilization Among Reproductive-Age Women Residing in a Rural Maternity Care Desert. J Community Health. 2021;46:108–16. 10.1007/S10900-020-00852-6/METRICS. 10.1007/s10900-020-00852-6 32488525

[19] Kozhimannil KB, Leonard SA, Handley SC, Passarella M, Main EK, Lorch SA (2023). Obstetric Volume and Severe Maternal Morbidity Among Low-Risk and Higher-Risk Patients Giving Birth at Rural and Urban US Hospitals. JAMA Health Forum.

[20] Minion SC, Krans EE, Brooks MM, Mendez DD, Haggerty CL (2022). Association of Driving Distance to Maternity Hospitals and Maternal and Perinatal Outcomes. Obstetrics and Gynecology.

[21] American Heart Association. Target: Stroke-When Seconds Count. https://www.heart.org/en/professional/quality-improvement/target-stroke/learn-more-about-target-stroke. Accessed 24 Jan 2024.

[22] Nageotte MP, Vander Wal B (2012). Achievement of the 30-minute standard in obstetrics can it be done?. Am J Obstet Gynecol.

[23] Boehm FH (2012). Decision to incision: Time to reconsider. Am J Obstet Gynecol.

[24] United States Census Bureau. “S1301: Fertility.” American Community Survey, 2017-2021. 2023. https://www.census.gov/programs-surveys/acs/data.html.

[25] National Center for Health Statistics. Final Natality Data. 2021. https://www.cdc.gov/nchs/data_access/vitalstatsonline.htm.

[26] U.S. Census Bureau. Why We Ask Questions About Race.https://www.census.gov/acs/www/about/why-we-ask-each-question/race/ (accessed 31 Mar. 2024).

[27] Manson S, Schroeder J, Van Riper D, Knowles K, Kugler T, Roberts F, et al. IPUMS National Historical Geographic Information System: Version 18.0 [Centers of Population GIS File, 2017-2021]. Minneapolis, MN. 2023. https://data2.nhgis.org/main.

[28] United States Census Bureau. TIGER/Line Shapefiles. 2021. https://www.census.gov/geographies/mapping-files/time-series/geo/tiger-line-file.2021.html#list-tab-790442341.

[29] American Hospital Association, Hospital Data. 2021. https://www.ahadata.com/aha-annual-survey-database.

[30] American Hospital Association. About the AHA https://www.aha.org/about (accessed 24 Jan. 2024).

[31] Interrante JD, Carroll C, Handley SC, Kozhimannil K. An Enhanced Method for Identifying Hospital-Based Obstetric Unit Status. University of Minnesota Rural Health Research Center. 2022. https://rhrc.umn.edu/wp-content/uploads/2023/04/UMN-OB-Unit-Identification-Methods_4.14-update.pdf.

[32] Centers for Medicare and Medicaid. Provider of Service Files- Hospital & Non-Hospital Facilities. 2021. https://data.cms.gov/provider-characteristics/hospitals-and-other-facilities/provider-of-services-file-hospital-non-hospital-facilities/data/q4-2021.

[33] March of Dimes. 2023 Maternity Care Deserts Report Technical Notes. 2023. https://www.marchofdimes.org/peristats/assets/s3/reports/documents/MaternityCareDesertsReport-TechnicalNotes.pdf (accessed 31 Mar. 2024).

[34] U.S. Health Resources and Services Administration (HRSA). Area Health Resources Files. 2022. https://data.hrsa.gov/topics/health-workforce/ahrf.

[35] Peterson LE, Fang B, Phillips RL, Avant R, Puffer JC (2019). The American Board of Family Medicine’s Data Collection Method for Tracking Their Specialty. J Am Board Family Med.

[36] American Association of Birth Centers. 2021. https://www.birthcenters.org/.

[37] Economic Research Service US Department of Agriculture. Urban Influence Codes. 2021. https://www.ers.usda.gov/data-products/urban-influence-codes/.

[38] Environmental Systems Research Institute (ESRI). ArcGIS Pro version 3.0. 2022. https://www.esri.com/en-us/arcgis/products/arcgis-pro/overview.

[39] ESRI. What is the ArcGIS Network Analyst extension? https://pro.arcgis.com/en/pro-app/latest/help/analysis/networks/what-is-network-analyst-.htm (accessed 24 Jan. 2024).

[40] SAS. Version 9.4 Cary, NC: SAS Institute Inc; 2020. https://www.sas.com/en_us/home.html.

[41] ESRI. Optimized Hot Spot Analysis (Spatial Statistics). https://pro.arcgis.com/en/pro-app/3.1/tool-reference/spatial-statistics/optimized-hot-spot-analysis.htm#:~:text=This%20tool%20identifies%20statistically%20significant,multiple%20testing%20and%20spatial%20dependence. (accessed 31 Mar. 2024).

[42] Brantley MD, Davis NL, Goodman DA, Callaghan WM, Barfield WD (2017). Perinatal regionalization: a geospatial view of perinatal critical care, United States, 2010–2013. Am J Obstet Gynecol.

[43] Weiss AJ, Roemer M, Pickens GT. Methods for Calculating Patient Travel Distance to Hospital in HCUP Data. HCUP Methods Series Report US Agency for Healthcare Research and Quality 2021.

[44] Handley SC, Passarella M, Herrick HM, Interrante JD, Lorch SA, Kozhimannil KB (2021). Birth Volume and Geographic Distribution of US Hospitals with Obstetric Services. JAMA Netw Open.

[45] Shabo V, Friedman H. Distances to Hospital-Based and Skilled Nursing Care Make Paid Leave Critical for Rural Communities. New America. 2022. https://www.newamerica.org/better-life-lab/reports/health-work-and-care-rural-america/distances-to-travel-to-hospital-based-health-care/.

[46] Hung P, Casey MM, Kozhimannil KB, Karaca-Mandic P, Moscovice IS (2018). Rural-urban differences in access to hospital obstetric and neonatal care: how far is the closest one?. J Perinatol.

[47] Guo J, Hernandez I, Dickson S, Tang S, Essien UR, Mair C (2022). Income disparities in driving distance to health care infrastructure in the United States: a geographic information systems analysis. BMC Res Notes.

[48] Walther F, Kuester D, Bieber A, Malzahn J, Rüdiger M, Schmitt J (2021). Are birth outcomes in low risk birth cohorts related to hospital birth volumes? A systematic review. BMC Pregn Childbirth.

[49] Thorsen ML, Harris S, Palacios JF, McGarvey RG, Thorsen A (2023). American Indians travel great distances for obstetrical care: Examining rural and racial disparities. Social Science and Medicine.

[50] Centers for Disease Control and Prevention. Disparities and Resilience among American Indian and Alaska Native People who are Pregnant or Postpartum. 2022. https://www.cdc.gov/hearher/aian/disparities.html (accessed 25 Jan. 2024).

[51] Kozhimannil KB, Interrante JD, Tofte AN, Admon LK, Kozhimannil KB (2020). Severe Maternal Morbidity and Mortality Among Indigenous Women in the United States. Obstetrics and Gynecology.

[52] Haynes R, Jones AP, Sauerzapf V, Zhao H (2006). Validation of travel times to hospital estimated by GIS. Int J Health Geogr.

[53] Delamater PL, Messina JP, Shortridge AM, Grady SC (2012). Measuring geographic access to health care: raster and network-based methods. Int J Health Geogr.

[54] Phibbs CS, Luft HS (1995). Correlation of travel time on roads versus straight line distance. Med Care Res Rev.

[55] Sonenberg A, Mason DJ (2023). Maternity Care Deserts in the US. JAMA Health Forum.

[56] Stoll K, Titoria R, Turner M, Jones A, Butska L (2023). Perinatal outcomes of midwife-led care, stratified by medical risk: a retrospective cohort study from British Columbia (2008–2018). Can Med Assoc J.

[57] The White House. White House Blueprint for Addressing the Maternal Health Crisis. 2022. https://www.whitehouse.gov/wp-content/uploads/2022/06/Maternal-Health-Blueprint.pdf.

[58] Health Resources and Services Administration (HRSA). Rural Maternity and Obstetrics Management Strategies (RMOMS) Program. 2022. https://www.hrsa.gov/rural-health/grants/rural-community/rmoms. Accessed 25 Jan 2024.

[59] Health Resources and Services Administration (HRSA). What Is Shortage Designation? 2023. https://bhw.hrsa.gov/workforce-shortage-areas/shortage-designation#mcta (accessed 25 Jan. 2024).

[60] Tenorio B, Whittington JR (2023). Increasing Access: Telehealth and Rural Obstetric Care. Obstet Gynecol Clin North Am.

[61] Carroll C, Interrante JD, Daw JR, Kozhimannil KB (2022). Association Between Medicaid Expansion And Closure Of Hospital-Based Obstetric Services. Health Aff (Millwood).

[62] Searing A, Corcoran A, Alker J. Medicaid Expansion’s Effects on Families: More coverage, improved maternal health, better preventive care. Center For Children and Families 2021. https://ccf.georgetown.edu/2021/02/19/medicaid-expansions-effects-on-families-more-coverage-improved-maternal-health-better-preventive-care/ (accessed 25 Jan. 2024).

[63] World Health Organization (WHO). Human rights. https://www.who.int/news-room/fact-sheets/detail/human-rights-and-health (accessed 25 Jan. 2024).

