# Supplementary material for: Navigating geographical disparities: access to obstetric hospitals in maternity care deserts and across the United States
Source: BMC Pregnancy Childbirth. 2024 May 8;24:350. doi: 10.1186/s12884-024-06535-7 (PMC11080172; doi:10.1186/s12884-024-06535-7)
Supplement: Supplementary file 1 — Supplementary Material 1. [file 12884_2024_6535_MOESM1_ESM.docx]

**ADDITIONAL FILES**

File name: Additional File 1

File format: MS Word

Title of Data: State by state distance to obstetric care

Description of Data: Data for each state by maternity care designations and average distance and time to obstetric care.

| **State** | **Est. Births** | **Overall Average** | | **Full Access** | | **Limited Access** | | **Maternity Care Desert** | |
| --- | --- | --- | --- | --- | --- | --- | --- | --- | --- |
|  |  | **Miles** | **Time** | **Miles** | **Time** | **Miles** | **Time** | **Miles** | **Time** |
| Alabama | 58514 | 12 (9.9) | 17.8 (11.1) | 9.3 (7) | 14.7 (8) | 16.3 (11.6) | 22.5 (13) | 27.5 (10.7) | 34.9 (11) |
| Alaska | 10413 | 17.5 (69.4) | 60.6 (398.5) | 9.8 (21) | 19.8 (58.8) | |  | 254.4 (286) | 1316.7 (1821.6) |
| Arizona | 88808 | 8.1 (9.2) | 13.3 (10.7) | 7.8 (8.3) | 12.8 (9.9) | 21.2 (19.9) | 27.7 (21.6) | 47.8 (1.4) | 52.7 (2.6) |
| Arkansas | 37634 | 12.3 (11.4) | 17.7 (12.9) | 8 (7.5) | 13.3 (8.8) | 17.5 (12.7) | 22.2 (14) | 27 (10.6) | 34.2 (12.8) |
| California | 461770 | 6 (7.2) | 11.1 (8.5) | 5.9 (7.1) | 11 (8.4) | 8.2 (7) | 13.4 (9.5) | 31.5 (12.9) | 34.6 (10.4) |
| Colorado | 67425 | 7.5 (8.4) | 12.9 (9.8) | 6.6 (6) | 11.9 (7.4) | 17.9 (10.2) | 25.4 (12.2) | 46.7 (15.4) | 56.1 (18.4) |
| Connecticut | 35148 | 5.6 (4.4) | 11.5 (5.9) | 5.6 (4.4) | 11.5 (5.9) |  |  |  |  |
| Delaware | 9827 | 8.6 (6.2) | 15.2 (7.7) | 8.6 (6.2) | 15.2 (7.7) |  |  |  |  |
| Dist. of Columbia | 8375 | 2.6 (1.4) | 8.3 (2.9) | 2.6 (1.4) | 8.3 (2.9) |  |  |  |  |
| Florida | 227983 | 9 (8.3) | 15.6 (9.6) | 8.1 (6.5) | 14.7 (8.1) | 27.5 (15.4) | 33.4 (15.6) | 37.2 (11.8) | 46.2 (13.8) |
| Georgia | 132163 | 9.6 (7.2) | 15.7 (8.3) | 8.2 (5.6) | 14.3 (6.9) | 12.4 (8) | 18.4 (8.9) | 24.5 (9.2) | 31.3 (10.2) |
| Hawaii | 18827 | 10.8 (8.2) | 19.1 (12.5) | 10.8 (8.2) | 19.1 (12.5) | |  |  |  |
| Idaho | 23634 | 8.9 (10.4) | 14.2 (12) | 7.3 (8.1) | 12.6 (9.9) | 9.2 (7.3) | 14.2 (7.9) | 33.1 (16.7) | 39.4 (19.5) |
| Illinois | 153901 | 6.5 (7) | 11.9 (7.9) | 5.2 (4.8) | 10.6 (5.9) | 19.5 (9.8) | 25.8 (10.7) | 25.9 (8.5) | 32.3 (9.1) |
| Indiana | 82749 | 7.3 (6.5) | 12.8 (7.6) | 6.1 (4.8) | 11.5 (6) | 8.3 (6) | 13.6 (7.4) | 20.7 (8.9) | 27.4 (9.7) |
| Iowa | 40138 | 11.4 (10.2) | 16.3 (10.8) | 8.9 (8.6) | 13.7 (9.3) | 14.5 (8) | 20.1 (8.9) | 27.1 (6.7) | 32.1 (7.5) |
| Kansas | 40014 | 9.9 (10.5) | 14.6 (10.6) | 7.9 (8.1) | 12.7 (8.5) | 15 (10.9) | 19.2 (11.4) | 30.6 (10.9) | 34.2 (10.7) |
| Kentucky | 52740 | 11.6 (9.2) | 17.4 (10.2) | 8.2 (6.1) | 13.9 (7.4) | 15.9 (10) | 21.3 (10.8) | 23.8 (8.4) | 30.3 (9.5) |
| Louisiana | 58744 | 10.4 (9.5) | 16.3 (11) | 8.2 (7.2) | 13.8 (8.5) | 15.2 (8.5) | 22 (10.9) | 30.3 (10.3) | 37.7 (11) |
| Maine | 14208 | 11 (9.4) | 18.4 (12.6) | 11 (9.4) | 18.4 (12.6) | |  |  |  |
| Maryland | 75070 | 6.9 (5.1) | 13.1 (6.4) | 6.8 (5) | 13 (6.3) | 16.7 (4) | 26.5 (5.3) | 16.3 (6.5) | 23.9 (8.6) |
| Massachusetts | 75648 | 6.2 (4.9) | 13 (6.9) | 6.2 (4.9) | 13 (6.9) |  |  |  |  |
| Michigan | 117552 | 8.5 (8) | 13.6 (8.9) | 7.5 (6.6) | 12.6 (7.5) | 14.7 (9.6) | 20.6 (11) | 36.9 (12.3) | 44 (13.6) |
| Minnesota | 73901 | 8.2 (8.7) | 12.8 (9.6) | 7.5 (7.2) | 12.1 (7.9) | 12.2 (8.7) | 17.5 (9.3) | 27.9 (21.5) | 34.1 (24.4) |
| Mississippi | 38201 | 13.6 (12.5) | 19.1 (13.3) | 7.2 (5.5) | 12.5 (6.8) | 18.6 (13.5) | 24.7 (14.8) | 31.3 (9) | 36.9 (9.8) |
| Missouri | 77895 | 11.3 (10) | 16.6 (10.8) | 8 (6.9) | 13 (7.4) | 15.4 (10) | 20.7 (10.5) | 28.5 (8.9) | 34.9 (10.3) |
| Montana | 12418 | 14.7 (19.4) | 20.2 (20) | 10.7 (14.6) | 16.3 (16) | 59.1 (8.3) | 61.1 (10.2) | 52.1 (17.4) | 56.1 (16.9) |
| Nebraska | 26915 | 8.3 (8.8) | 12.9 (9.2) | 6.9 (6.7) | 11.6 (7.3) | 5.5 (5.1) | 10.4 (5.9) | 25 (12.6) | 28.6 (13) |
| Nevada | 36426 | 8 (12) | 13.1 (12.7) | 6.3 (8.9) | 11.4 (9.8) | 6.9 (7.1) | 11.9 (8.2) | 37.6 (20.1) | 43.8 (18.9) |
| New Hampshire | 14048 | 9.4 (8) | 16 (10.6) | 9.4 (8) | 16 (10.6) |  |  |  |  |
| New Jersey | 106279 | 5.4 (4.1) | 11.3 (5.6) | 5.3 (4.1) | 11.2 (5.6) | 7.7 (3.3) | 12.7 (4.4) |  |  |
| New Mexico | 25825 | 11.7 (13.3) | 17 (14.4) | 9.3 (9.6) | 15 (12.3) | 14.9 (12) | 19.1 (11.4) | 50.5 (22.5) | 52.6 (20.4) |
| New York | 224967 | 5.7 (6.4) | 12 (7.8) | 5.4 (6.1) | 11.8 (7.5) | 17.2 (9.3) | 23.9 (11.1) | 21.8 (6.4) | 30 (8.5) |
| North Carolina | 124231 | 9.5 (6.6) | 15.4 (8) | 8.4 (5.6) | 14.1 (7) | 13.3 (7.3) | 19.8 (8.8) | 22.8 (8.2) | 30.1 (10) |
| North Dakota | 11411 | 15.2 (21.1) | 19 (20.4) | 7.6 (12.6) | 11.4 (12.2) | 13 (14.3) | 18.3 (12.2) | 46.1 (21.2) | 49.1 (20.1) |
| Ohio | 146441 | 8.5 (6.7) | 14.2 (7.8) | 7.3 (5.1) | 12.8 (6.1) | 13.7 (10.1) | 19.8 (11.2) | 21.3 (6.9) | 28.5 (8.5) |
| Oklahoma | 52709 | 10.8 (11.2) | 15.5 (11.6) | 6.4 (5.2) | 11.1 (6.1) | 15 (13.5) | 19.9 (13.7) | 28.5 (11.6) | 33.1 (11.4) |
| Oregon | 46923 | 7.2 (7.9) | 13.3 (10.3) | 7.1 (7.7) | 13.2 (10) | 25.2 (7.5) | 29.8 (7.9) | 63.4 (23.9) | 89.8 (44.1) |
| Pennsylvania | 145623 | 8.5 (7.3) | 15.7 (9) | 8 (6.7) | 15 (8.4) | 15.7 (9.1) | 23.8 (11.7) | 25.8 (8.4) | 36.2 (11) |
| Rhode Island | 11821 | 5.9 (4) | 12.4 (5.6) | 5.9 (4) | 12.4 (5.6) |  |  |  |  |
| South Carolina | 63525 | 10.3 (7.5) | 16.7 (9.1) | 9.4 (6.8) | 15.7 (8.3) | 12.8 (8.5) | 19.6 (10) | 20.1 (10.4) | 27.5 (12.8) |
| South Dakota | 11840 | 13.6 (16.1) | 18.7 (17.4) | 9.1 (11.8) | 13.9 (12.4) | 5.6 (6.2) | 9.3 (7.5) | 35.4 (15.8) | 41.8 (18.3) |
| Tennessee | 82463 | 10.3 (8) | 16.5 (9.5) | 8.4 (6.2) | 14.4 (7.4) | 14.7 (9.7) | 21.3 (11.3) | 23.9 (7.8) | 32.3 (9.5) |
| Texas | 401736 | 8.5 (8) | 13.5 (8.1) | 7.1 (5) | 12.2 (5.8) | 17.3 (13.2) | 21.4 (13.4) | 30.4 (12.3) | 33.1 (11.4) |
| Utah | 49687 | 6 (6.5) | 10.6 (7.7) | 5.8 (5.4) | 10.4 (6.6) | 5.4 (10.7) | 9.8 (11.9) | 31.5 (19.3) | 36.7 (25.7) |
| Vermont | 5492 | 10.3 (7.6) | 17.3 (10.1) | 10.3 (7.6) | 17.2 (10) |  |  | 21.8 (7.7) | 33.8 (10) |
| Virginia | 99799 | 8.6 (8.1) | 14.7 (9.6) | 7.3 (6.5) | 13.2 (7.8) | 8.4 (6.9) | 14.8 (7.8) | 22 (12.1) | 30.2 (14.7) |
| Washington | 96266 | 7.6 (7) | 14.2 (10) | 7.4 (6.6) | 13.9 (9.5) | 8.1 (5.6) | 14.1 (7.9) | 20.8 (16.9) | 31.3 (21.9) |
| West Virginia | 18604 | 16.1 (15.1) | 23.2 (17.5) | 12.1 (13) | 18.3 (14.6) | 20.7 (16.4) | 27.6 (17.3) | 28.8 (13.8) | 39.9 (17.6) |
| Wisconsin | 66755 | 8.2 (7.7) | 13.1 (8.9) | 7.4 (6.8) | 12.1 (7.9) | 14.1 (9.9) | 19.6 (11.2) | 20.9 (10.3) | 27.9 (11.2) |
| Wyoming | 7909 | 11.6 (17.7) | 16.4 (18.4) | 8.4 (11.7) | 13.4 (13.6) | 7.7 (10.3) | 12.1 (15.9) | 58.2 (22.6) | 59.2 (22.6) |
| Puerto Rico | 20999 | 7 (5.2) | 13.9 (9) | 5.5 (4.9) | 11.8 (8.6) | 8.5 (3.9) | 15.7 (6.6) | 10.9 (4.6) | 19.4 (8.8) |
